# Yellow Himalayan Raspberry (*Rubus ellipticus* Sm.): Ethnomedicinal, Nutraceutical, and Pharmacological Aspects

**DOI:** 10.3390/molecules28166071

**Published:** 2023-08-15

**Authors:** Ananda Lamichhane, Gopal Lamichhane, Hari Prasad Devkota

**Affiliations:** 1 Collage of Pharmacy, Kyungpook National University, Daegu 41566, Republic of Korea; aagyan2018@knu.ac.kr; 2 Department of Nutritional Sciences, Oklahoma State University, Stillwater, OK 74078, USA; lamichhanegopal1@gmail.com; 3 Graduate School of Pharmaceutical Sciences, Kumamoto University, 5-1 Oe-honmachi, Chuo-ku, Kumamoto 862-0973, Japan; 4 Headquarters for Admissions and Education, Kumamoto University, Kurokami, 2-39-1, Chuo-ku, Kumamoto 860-8555, Japan; 5 Pharmacy Program, Gandaki University, Pokhara 33700, Nepal

**Keywords:** *Rubus ellipticus*, yellow Himalaya raspberry, ethnomedicine, bioactive chemical, anti-oxidant, anti-proliferative, Himalayan wild fruits

## Abstract

Yellow Himalayan raspberry (*Rubus ellipticus* Sm., Rosaceae) is a native species of the Indian subcontinent, Southern China, and the Philippines, which has been historically used as a traditional medicine and food. All of the parts of this plant have been used in traditional medicine to treat respiratory ailments, diabetes, and gastrointestinal disorder, and as an anti-infective agent. The scientific evaluation revealed a richness of macronutrients, micronutrients, and minerals in the fruits, indicating its potential use as a nutraceutical. Furthermore, this plant has been found to be rich in various secondary metabolites, including polyphenols, flavonoids, anthocyanins, tannins, and terpenoids. Ascorbic acid, kaempferol, gallic acid, and catechin are some of the compounds found in this plant, which have been widely discussed for their health benefits. Furthermore, various extracts and compounds obtained from *R. ellipticus* have shown antioxidant, antidiabetic, anticancer, anti-inflammatory, nephroprotective, antipyretic, anticonvulsant, and anti-infective activities investigated through different study models. These findings in the literature have validated some of the widespread uses of the fruits in folk medicinal systems and the consumption of this nutritious wild fruit by local communities. In conclusion, *R. ellipticus* holds strong potential for its development as a nutraceutical. It can also improve the nutritional status of villagers and uplift the economy if properly utilized and marketed.

## 1. Introduction

Medicinal plants serve as an integral component of healthcare in large populations throughout the world. The incorporation of medicinal plants in traditional medicine, spices, nutraceuticals, and as a source of lead compounds has helped humanity fight against many health ailments. Only a fraction of plants have been scientifically documented and studied, and there are many higher plants that are yet to be discovered [1,2]. This diversity of plants and secondary metabolites present in these plants indicates tremendous possibilities for drug discovery and development [3,4]. Currently, scientists are more inclined toward investigating and gathering scientific evidence of ethnomedicinal plants to discover new lead compounds [5,6,7]. This approach, on one hand, would help validate the use of these plants as traditional medicine and nutraceuticals, and on the other hand, increases the likelihood of the discovery of lead compounds compared to random screening [8,9]. Furthermore, polyherbal formulation synergistic therapeutic effects and reduced adverse effects due to individual ingredients can be developed with low doses of multiple ingredients for the management of diseases [10,11,12]. Many bioactive compounds with beneficial effects for various chronic diseases such as diabetes [13], hypercholesterolemia [14,15], high blood pressure [16], cirrhosis [17,18], chronic obstructive pulmonary disease (COPD) [19,20], cancer [21], bacterial infections, aging-related degenerative diseases, and autoimmune diseases [22] have been identified. Research on natural products is still ongoing, searching for pharmacologically active extracts, herbal formulations, and, possibly, newer lead compounds by which to develop them as an effective therapeutic intervention [23]. The growing interest in plant-derived herbal medicine and nutraceuticals with bioactive polyphenols, terpenoids, vitamins, amino acids, and minerals is another reason for the growing research on natural products [24,25,26]. Although researchers have worked on a synthetic approach to develop various derivatives, such as amino sugars, iminosugars, and glycopeptides, as glycosidase inhibitors to discover potent antidiabetic compounds [27,28], the potential of plants as a source of lead compounds cannot be ignored. This is because there are still many unstudied plants that might contain such compounds, indicating the importance of in-depth studies on medicinal plants. Many plant-derived formulations have already been marketed; however, we still need to resolve the challenges regarding the identification of bioactive molecules, the depth study of their pharmacological properties, as well as the design of an effective formulation and drug delivery system using those herbal medicines as well [29,30].

**Figure 1:**
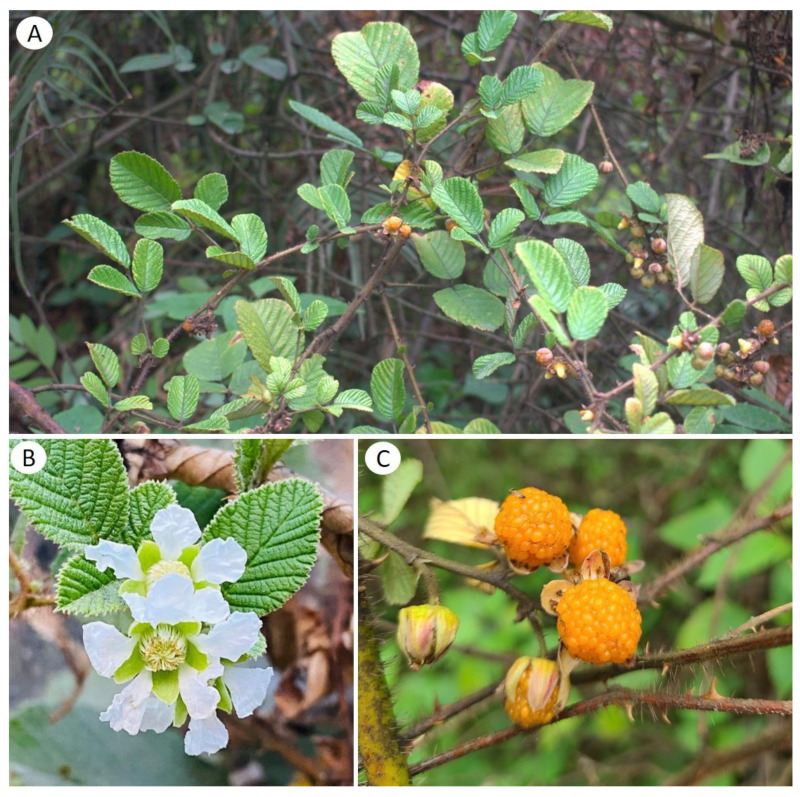
Photographs of different plant parts of *Rubus ellipticus* Sm. (**A**) Shrub, (**B**) flowers, and (**C**) fruits. (Photo credits: Dipa Paneru (**A**,**C**), Sujata Lamichhane (**B**).)

*Rubus ellipticus* Sm., commonly known as the yellow Himalayan raspberry (Figure 1), is an evergreen shrub belonging to the Rosaceae family. There are approximately 600–800 species belonging to the *Rubus* genus, categorized into 12 sub-genera [31,32]. Although different species of the *Rubus* genus are cultivated worldwide, *R. ellipticus* is the most abundant and wild-growing fruit species on the Indian subcontinent [33]. It is native to the Indian subcontinent, Southern China, and the Philippines, and it is also distributed in Thailand, Sri Lanka, Jamaica, Ecuador, as well as the Central Coast of Australia [34,35,36]. It is locally called “Ainselu” in Nepal and “Hisalu” in India [31,37] and has been reported to be distributed in the hilly and Himalayan region at an altitude of 2600 m. It is a shrub of about 1–3 m in height, covered with glandular hair and a needle-like hook on its stem. The plant is well known for its golden-yellow subglobose fruit, with high nutritional value. Upon maturity, the fruit changes from a green to golden or yellow color, increasing both its esthetic and medicinal value. In Tibetan medicine, matured fruits of *R. ellipticus* are used to treat coughs and fever [32]. Its roots and shoots are well-known renal tonic, anti-diuretic agents. Recently, this plant has been referred to as a high-nutrient food, and dried fresh leaves are used in powders and other forms. The leaves are rich in many bioactive compounds, including phenols [38], amino acids [39,40], flavonoids [38,41], and terpenoids [42]. In this study, we aim to examine all of the traditional and modern uses of *R. ellipticus*, including its ethnomedicinal uses, nutritional value, bioactive chemical constituents, and pharmacological activities reported in the literature.

## 2. Methods 

More than 100 research publications were used as the primary source of data for this review paper. Original research articles, review papers, books, book chapters, reports, and short communications were used as the data source from different database sites like Google Scholar, Scopus, and Web of Science. Various keywords such as ethnomedicinal uses of *Rubus ellipticus*, in vitro and in vivo studies of *R. ellipticus*, chemical constituents of *R. ellipticus*, nutritional importance of *R. ellipticus*, and different pharmacological properties of *R. ellipticus* were used to search the required information for the database. Ethnomedicinal uses, bioactive chemical constituents, and nutritional composition were especially taken into consideration and the reported pharmaceutical activities of such contained bioactive chemicals were extracted from the database to prepare this manuscript. 

## 3. Botanical Description and Distribution

*Rubus ellipticus* is a shrub about 1–3 m tall with purplish brown or brownish branches, covered in sparse, curved prickles and dense, purplish brown bristles or glandular hairs. The leaves are composed of three leaflets, with the terminal leaflet having a longer petiole than the lateral leaflets, and the petiole is 2–6 cm long. The leaves have stipules that are linear and 7–11 mm long, and the blade of the leaflets is elliptic or obovate, with the terminal leaflet being larger than the lateral leaflets. The inflorescences are terminal, with dense glomerate racemes that have several (10 or more) flowers. The flowers are 1–1.5 cm in diameter, bisexual, and have white or pink petals that are longer than the sepals. The aggregate fruit is golden yellow and subglobose, with a diameter of approximately 1 cm. It blooms from March to April and bears fruit from April to May [43,44,45].

It grows quickly in both open, sunny areas and dense rainforests and thrives in moist forests in the high-altitude forests of the Himalaya region [46]. It grows along highways, hillsides, thickets, slopes, mountain valleys, and sparsely populated woodlands between 300 and 2600 meters above sea level in an area with annual rainfall between 2000 and 6500 mm [47]. The plant is commonly found in Southern Asia, Southwestern China, Myanmar, Bhutan, Laos, Pakistan, Sri-Lanka, Nepal, Philippines, Vietnam, Thailand, and India. The plant is also found in a certain area of Australia’s Central Coastal New South Wales, Southern Queensland [47] and some countries of Africa [48]. Within India, it is found in Assam, Sikkim, Tamil Nadu, Kerala, and Maharashtra states [32].

## 4. Ethnomedicinal Uses

The Rubus genus offers opportunities for the farmers not only economically but also medicinally. Its fruits provide extra income to farmers in rural areas without investment, thereby helping to uplift their economic status [49]. According to Tibetan traditional medicine, the leaves and fruits of *R. ellipticus* can be used treat a variety of conditions such as bronchitis, nausea, ulcers, and diabetes, as well as for its antimicrobial, carminative, and tonic properties [37,50,51]. The root bark is used to treat diarrhea, dysentery, fractured bones, and as an abortifacient and emmenagogue [52]. The shoot of the plant is sometimes chewed to soothe stomach discomfort, and a root decoction can be consumed to warm the stomach [53,54,55,56]. This plant comes under the top ten wild edible medicinal plants of the Tanahun District of Nepal, owing to the widespread use of its fruits and root in folk medicine. Additionally, the juice of *R. ellipticus* is used for making squash and jams because of its flavor and color [50]. Details of ethnomedicinal uses of *R. ellipticus* are mentioned in Table 1.

**Table 1:** Ethnomedicinal uses of *R. ellipticus* Sm.

Parts of Plants Used	Dose/Formulation	Used in Disease	References
Bark	Bark juice is consumed	Common cold and blood disorders	[57]
	Paste mixed with water and consumed	As an antidiuretic and renal tonic	[32,55]
	-	Common cold	[57,58]
Fruit	Raw fruit is consumed	Abdominal pain	[59]
	Decoction	Dysentery	[60]
	Juice is consumed	Diabetes	[61,62]
	Eaten raw	Diarrhea and as diuretics	[63]
	Juice is consumed	Sore throat and cold	[64]
	Juice is consumed	Cardiac and blood-related diseases	[62]
	10–20 g fruits, 3 times a day	Gastritis, antacid, diarrhea, and dysentery	[65]
	Juice is consumed	Indigestion	[66]
	Juice is consumed	Fever and cough	[60]
Ripe fruit	1 Teaspoon decoction 3 times a day	Food poisoning	[67,68]
	Decoction	Loss of appetite, general debility, continuous vomiting after eating	[67,68]
	Eaten raw	As an aperient	[69,70]
	-	Constipation	[65]
	Taken as juice and in raw form	Mouth ulcer	[69]
Leaves	Juice of 20 leaflets consumed	As a febrifuge	[67,68]
	Juice/powdered is consumed	vomiting	[59]
	Juice is consumed	Mouth ulcer and gastrointestinal disorders	[69,70]
Leaves and fruits	Consumed as juice, powder, or in raw form	Gastrointestinal problems and mouth disorders	[71]
Root	5–10 g of the crushed root as a juice	Used to reduce fever	[67,68]
	Paste	Applied on wound	[60,72]
	Powder/Juice	Fever and diarrhea	[73,74]
	Root paste is mixed with various other plants and 1 spoon (fresh) or 1/2 spoon (dry) is consumed with 1 glass of water once a day	Mental disorder	[64]
	Paste applied as a poultice	Paralysis, Bone facture	[50,64,75]
	Paste applied as a poultice	Colic pain, aggression	[75]
	Root paste and ash of *Eleusine coracana* mixed and applied externally, once a day	Wound healing	[76]
	Juice is taken	Gastrointestinal and respiratory problems	[77]
	Decoction is prepared together with other plants and consumed	Typhoid and stomach pain, Respiratory tract infection, Gastrointestinal tract infection	[77]
	Decoction is consumed	Used to kill stomach worms and cure other gastric problems	[74]
	Powder	Rhinitis and sinusitis	[77]
	10–20 mL of juice taken	Diarrhea, cholera, gastritis, sore throat	[78]
	Decoction is consumed	Typhoid fever	[79]
	Juice is consumed	Urinary tract infection	[57]
Root and fruit	Eaten raw	Excessive thirst and weakness	[73]
Root and shoot	-	Colic pains, antiprotozoal activity against *Entamoeba histolytica*, hypoglycemic activity	[64]
Root and young shoots	Paste is taken orally	Throat pain	[80]
Whole part	Raw/Juice is consumed	Hypothermia	[75,81]
	Raw/Juice is consumed	As an astringent and tonic	[82]
	-	Epilepsy	[83]
	Crushed plant parts along with *Osbeckia nepalensis* is applied with to skin	Dermatitis	[77]

## 5. Nutritional Composition

Many studies have reported macronutrients such as carbohydrates, protein, fat, fiber, and several minerals such as calcium, phosphorus, and copper on fruits of the *Rubus* species. Table 2 and Table 3 below show a comparative representation of the profile of macronutrients and micronutrients of *R. ellipticus,* respectively. Values for *R. fruticosus,* another species of *Rubus*, are also provided, but some of these values are for residues after extracting juice. However, it should be noted that the nutritional composition, total phytochemical content, and compounds isolated vary based on various factors such as environmental factors, ripening stage, processing after collection such as drying, extraction solvents and methods, and analysis methods [84,85,86,87,88,89].

## 6. Phytochemical Constituents

**Figure 2:**
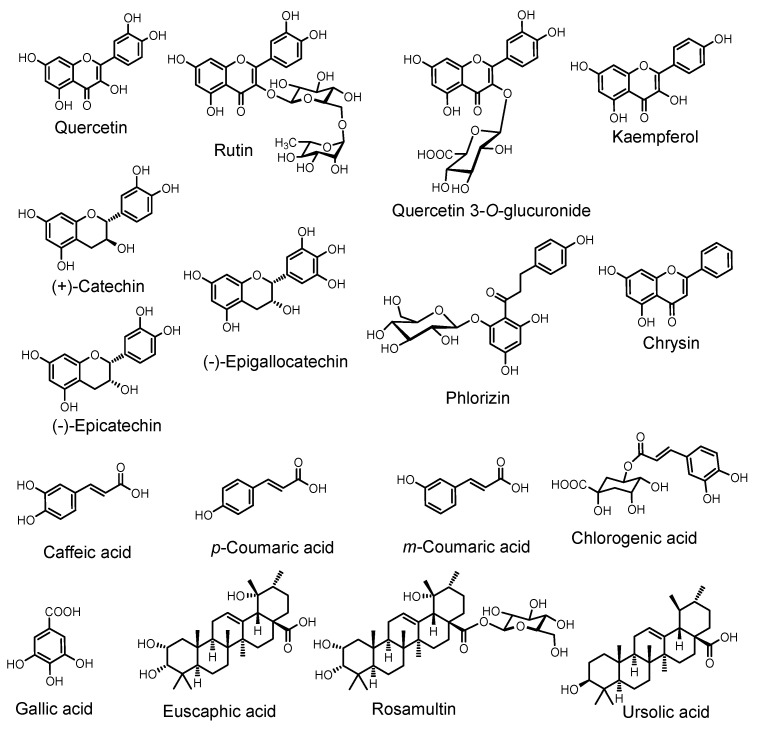
Chemical structure of major phytochemicals found in *R. ellipticus* plant.

*Rubus* species possess diverse secondary metabolites including phenolics, flavonoids, anthocyanins, and tannins. Table 4 shows the values of total phenolic, flavonoid, anthocyanin, and tannin contents observed in *R. ellipticus* and *R. fruticosus*. The main compounds isolated/identified from *R. ellipticus* are listed in Table 5 and structures of main compounds are provided in Figure 2. With respect to another species of *Rubus*, the main compounds reported from *R. fruticosus* were campesterol, rubinic acid, rubitic acid, kaempferol, stigmasterol, matairesinol, tocopherol, morin, etc. [90,96].

**Table 4:** Major phytochemicals contents present in *R. ellipticus* and *R. fruticosus*.

Phytochemicals	Value for *R. ellipticus*	Value for *R. fruticosus*
Total phenol content	343.75 ± 2.21 µg GAE/mg [35]	412.38 ± 18.78 mg GAE/100 g fresh weight [97]
Total flavonoid content	433.5 ± 13.39 mg CE/100 g [41]	77.77 mg QE/100 g [31]
Total anthocyanin content	1.71 ± 0.08 CGE/100g [98]	152 mg/100 g [31]
Total tannin content	628.32 ± 3.17 mg TAE/g [31]	6.50 ± 3.1% of dry matter [99]

µg GAE/mg: Microgram gallic acid equivalent per milligram of extract; mg CE/100 g: milligram catechin.

**Table 5:** List of bioactive chemical constituents isolated from *R. ellipticus*.

Phytochemical Classes	Bioactive Compound	Plant Part	References
Flavonoids/Chalcones	Quercetin	Fruit	[100]
	Rutin	Fruit	[85]
	Quercetin 3-*O*-glucuronide	Fruit	[101]
	Phloridzin	Fruit	[85,102]
	Kaempferol	Leaves	[38,103]
	Catechin Epicatechin Epigallocatechin	Fruit	[104,105,106]
	Chrysin Cyanidin Pelargonidin	Fruit	[107]
Phenolic acids/Organic Acids	Gallic acid	Fruit and Leaves	[31,41]
	Malic acid	Fruit	[64]
	Ellagic acid	Fruit and Leaves	[38,102]
	Chlorogenic acid	Fruit and Leaves	[31]
	Citric acid	Fruit	[32]
	Ascorbic acid	Fruit	[32,98]
	Acuminatic acid	Root	[108]
	Quinic acid	Fruit	[64]
	Caffeic acid	Leaves and Fruit	[31,41]
	*m*-Coumaric acid	Fruit	[56]
	*p*-Coumaric acid	Fruit	[56,85]
Ellagitannins	Lambertianin C Sanguiin H6	Fruit	[101]
Triterpenes and Sterols	Tormentic acid Miquelianin	Leaves and Root	[34,102]
	Euscaphic acid	Root	[42]
	*β*-Sitosterol *β*-Sitosterol-*β*-D-glucoside	Leaves and Root	[102,109]
	*β*-Carotene	Fruits and Leaves	[33,85]
	Rosamutin Sericic acid Buergericic acid	Root	[42]
	Oleanane	Leaves	[110]
	Ursolic acid	Root	[108]
	Campesterol	Leaves and Root	[34,102]
	Niga-ichgoside-F1 [28-*β*-Glucopyranosyl ester of 19 *α*-hydroxyasiatic acid]	Leaves and Fruits	[111]
	Octacosanol Octacosanic acid	Root	[53,102]
	3-*β*-Hydroxy-urs-12,18-diene-28-oic-acid-3-*O*-(*β*-D-glucopyranosyl (1-4)-*α*-*L*-arabinopyranoside	Fruit and Aerial Part	[53,64]
	24-Deoxysericoside	Whole Plant	[111]
Amino acids	Tyrosine Hydroxy proline Serine Histidine Leucine	Fruit	[39]

## 7. Biological Activities

### 7.1. Antioxidant Activity 

*Rubus* species are enriched with various bioactive phytochemicals, especially anthocyanin, phenolic, and flavonoid compounds. All these components play an important role in combating various diseases by scavenging reactive oxygen species (ROS) [35,112]. There are various mechanisms through which *R. ellipticus* exhibits its antioxidant potential depending upon the bioactive chemical constituents present on the plant. These include scavenging free radicals and ROS, thereby reducing oxidative stress and cellular damage, neutralizing the free radicals by donating electrons, quenching singlet oxygen, and chelation of the metal ions [113]. According to Badhani et al., a high level of various types of chemical constituents including 19.80 mg/100 g fresh weight (fw) of ascorbic acid and 0.99 mg/100 g fw of *β*-carotene contributes to the antioxidant properties of *R. ellipticus* [33]. For instance, half maximum inhibitory concentration (IC_50,_) values of *R. ellipticus* fruit extract from the *α*, *α*-diphenyl-*β*-picrylhydrazyl (DPPH) assay, ferric ion reducing antioxidant power (FRAP) assay, and 2,2′-azino-bis(3-ethylbenzothiazoline-6-sulfonic acid) (ABST) assay were found to be 32.8 ± 0.5 µg/mL, 73.1 ± 0.5 µM Fe^2+^ equivalent, and 39.2 ± 1.1 µg/mL, respectively, revealing the strong antioxidant property of *R. ellipticus* [114]. Likewise, George et al. reported the significant potential of methanolic leaf extract to scavenge DPPH free radicals with an IC_50_ value of 6.96 ± 2.32 µg/mL, which was comparable to standard BHA, BHT, and quercetin with IC_50_ values of 4.88 ± 1.45 µg/mL, 13.18 ± 1.43 µg/mL, and 4.12 ± 1.67 µg/mL, respectively [115].

### 7.2. Antimalarial Activity 

Sachdeva et al. reported the potent anti-malarial activity of *R. ellipticus* leaf extract in both in vitro and in vivo models. From an in vitro study, they found the antimalarial efficacy of *R. ellipticus* with an IC_50_ value of 14.26 μg/mL against the *Plasmodium falciparum* INDO strain (PfINDO). They observed 64% of inhibition against *Plasmodium berghei* at a dose of 500 mg/kg [80]. Another study using silver nanoparticles of the leaf extract of *R. ellipticus* also showed an antimalarial effect against the malarial vector *Anophelese stephensi* [116]. They found that the aqueous-extract-loaded silver nanoparticles showed greater larvicidal, ovicidal, oviposition deterrent, and adulticidal activities against *Culex quinquefasciatus*, *Anophelese stephensi*, and *Aedes aegypti* as compared to the leaf extract alone [116].

### 7.3. Antidiabetic Activity

*α*-Amylase and *α*-glucosidase are the carbohydrate-hydrolyzing enzymes that catalyze the breakdown of starch and disaccharides into glucose and are thus important in regulating blood glucose level [117,118]. Subba et al. reported a moderate *α*-amylase inhibitory activity of the methanolic leaf extract with an IC_50_ value of 269.94 ± 0.11 µg/mL [37]. Li et al. isolated 31 triterpenoids present in *R. ellipticus* and performed their *α*-glucosidase inhibition assay. They found that, among all the extracted compounds, euscaphic acid was found to be the most potent with an IC_50_ value of 0.65 ± 0.09 mM, which was comparable with the positive control, acarbose (0.82 ± 0.11 mM) [42]. Sharma et al. performed an antidiabetic activity assay of *R. ellipticus* fruit extract using the glucose tolerance test and alloxan-induced diabetes assay using two different experimental diabetic models (Swiss albino mice and Wistar albino rats). It was reported that the ethanolic extract of the fruit was more effective in reducing the blood glucose level in alloxan-induced diabetic Wistar albino rats and Swiss albino mice as compared with its ether and aqueous extract [119]. Since the ethanolic extract was found to contain triterpenoids, flavonoids, as well as saponins, these compounds might contribute to the higher antidiabetic activity of the ethanolic extract compared to others [42,118,120,121].

### 7.4. Antiproliferative and Anticancer Activity 

Uncontrolled proliferation or production of the cells results in cancer. Recently, most researchers have been focused on either synthesizing new compounds or searching potent natural compounds that can be used for the treatment of cancer. A study conducted by George et al. showed an increased survival in tumor-enriched Swiss albino rats with the administration of 250 mg/kg of methanolic leaf extract from *R. ellipticus*. A similar finding was observed in another study showing a reduced solid tumor volume of Dalton’s Lymphoma Ascites-induced Swiss albino mice when treated with 100 mg/kg of *R. ellipticus* methanolic leaf extract [115]. An in vitro study by Saini et al., against two human cervical cancer cell lines (C33A and HeLa), showed reduced cell proliferation upon treatment with fruit extract. They found a 60% inhibition in proliferation of the C33A cell line by methanol and acetone extract and the complete inhibition of proliferation in the HeLa cell, with no toxicity to the normal cells [41,122]. The antiproliferative activity of *R. ellipticus* extract might be due to the presence of the higher concentration of gallic acid and ellagic acid in its extract [122,123]. Different extracts of *R. ellipticus*, *R. niveus*, and *R. fairholmianus* also showed a potent antiproliferative activity of human colon cancer cells (Caco-2) in the MTT ((3-(4,5-dimethylthiazol-2-yl)-2,5-diphenyl-2H-tetrazolium bromide) assay [38]. This might be due to the presence of diverse phytochemicals in the fruit extract such as kaempferol [38,124].

### 7.5. Anti-Inflammatory Activity 

Parimelazhagan et al. reported the anti-inflammatory activity of *R. ellipticus* methanolic leaf extract using carrageenan-induced inflammation and croton-oil-induced ear inflammation models in rats. The supplementation of methanolic leaf extract at doses of 400 mg/kg and 200 mg/kg to carrageenan-induced inflamed rats showed a reduction in inflammation. The extract showed a reduction in rat paw with edema by about 66.47% and 45.78%, respectively, at those doses, which were comparable to the standard drug, indomethacin [46]. Hydroalcoholic (90% ethanol) extract of the root of *R. ellipticus* significantly inhibited the inflammation induced by the carrageenan in rats. The extract was able to reduce the vascular permeability induced by carrageenan, resulting from reduced accumulation of the fluid in the vascular tissues, lowering inflammation [50].

### 7.6. Antifertility Activity 

Prakash et al. performed a series of screening in order to identify the ability of the ethanolic extract of the *R. ellipticus* plant for its anti-fertility properties. They observed that the extract of *R. ellipticus* possessed a potent antifertility effect with estrogenic action, as evidenced from screening using rats, mice, and hamsters [125]. Likewise, Dhanabal et al. reported 91.43% of anti-fertility activity, 37.10% of early abortifacient activity, and 54.33% of anti-implantation activity, when 200 mg/mL of *R. ellipticus* leaf extract was administered to female Wistar albino rats. Additionally, an anti-implantation effect was also observed in female albino rats due to the significantly increased reabsorption site and decreased implantation sites upon treatment with the extract [126]. Similarly, the anti-implantation activities of *R. ellipticus* root extract and the whole plant without the root extract were reported as 60% and almost 100%, respectively, in albino rats during 7 days of pregnancy [127].

### 7.7. Nephroprotective Activity 

Sharma et al. studied the nephroprotective activity of various extracts of *R. ellipticus* (petroleum ether, ethanolic, aqueous). These extracts were able to normalize increased blood urea nitrogen, serum uric acid, creatinine, and serum urea levels induced by cisplatin and gentamicin [128]. Such a potent nephroprotective activity might be due to the presence of certain phytochemicals like phenols, tannins, flavonoids, and triterpenoids, which act as an antioxidant, reducing the risk of renal dysfunction [129,130,131]. Likewise, the ethanolic extract of *R. ellipticus* was found to be an effective nephroprotective agent compared to its petroleum ether and aqueous extract. The extract also improved histological disturbance in acetaminophen-induced nephrotoxicity in animal models [110].

### 7.8. Antiviral Activity 

According to Panda et al., both aqueous as well as ethanol extracts of *R. ellipticus* showed potent antiviral as well as cytotoxic activities against enterovirus 71 strains revealed by various parameters like the selectivity index, selectivity surface, as well as therapeutic index [132].

### 7.9. Antipyretic Activity 

The antipyretic activity of the leaf methanolic extract of *R. ellipticus* was studied using yeast-induced hyperpyrexia in rats. Rectal temperature was continuously monitored after the treatment of 200 and 400 mg/kg doses of the extract. A reduction in elevated rectal temperature was observed in the 3rd to 7th hour after treatment, which was comparable to that of the standard drug, paracetamol. This suggested the ability of *R. ellipticus* extracts to inhibit various inflammatory cytokines produced during hyperpyrexia [46].

### 7.10. Effect on Central Nervous System (CNS) 

The anticonvulsant property of *R. ellipticus* ethanolic leaf extract was reported using maximum electroshock-induced convulsion in an experimental animal model [133]. In such studies, the severity of convulsion is assessed based on the duration of the flexion, extension, clonus, stupor, and recovery phase [134]. Administration of the ethanolic extract (100 mg/kg per oral) was found to inhibit the extensor phase of convulsion [32,133].

### 7.11. Antimicrobial Activity 

Panda et al. studied the antimicrobial effect of acetone, water, and ethanolic extracts of the leaf of *R. ellipticus* against *Escherichia coli*, *Staphylococcus aureus*, and *Candida albicans*. More than 50% of growth inhibition of all these microorganisms (bacteria and fungi) was shown by most of the extracts (acetone, water, ethanol) [132]. Likewise, Khanal et al. used disc diffusion and the Resazurin microtiter assay technique to test the methanolic extract of *R. ellipticus* root bark for antibacterial activity against Gram-positive *S. aureus*, Gram-negative *Klebsiella pneumoniae*, and *Salmonella typhi.* They found that the methanolic extract of *R. ellipticus* root bark has significant antibacterial activity against *S. aureus* with a zone of inhibition of 17 mm, but no impact was observed on Gram-negative organisms [135]. Singh et al. reported that the ethanolic leaf extract was capable of inhibiting *Pseudomonas aeruginosa* and *Enterococcus faecalis* at an extract concentration of 62.5 µg/mL, whereas *S. aureus* and *S. typhi* were sensitive at 125 µg/mL of crude extract [136]. *R. ellipticus* ethanolic fruit extract was also reported to have significant inhibition against *E. coli* and *Streptococcus pyogenes* [39]. Dhatwalia et al. studied the antimicrobial effect of cuprous-oxide-mediated nanoparticles of the fruit extract of *R. ellipticus*. They found that the synthesized nanoparticle can significantly inhibit Gram-positive bacteria (*Bacillus subtilis* and *S. aureus*) with minimum inhibitory concentrations (MICs) of 7.81 µg/mL and 15.62 µg/mL, respectively. Those nanoparticles also inhibited the growth of Gram-negative *P. aeruginosa* and *E. coli* with MICs of 31.25 µg/ mL and 31.25, µg/mL, respectively [114].

### 7.12. Wound Healing Activity 

George et al. studied the percentage of contraction of the wound via an excision and infection model with 1% and 2% *w*/*w* ointments of *R. ellipticus* methanolic leaf extract. In the excision model, a 2% acetone-extract-treated Wistar male rat demonstrated 94.23% of contraction on the 12th day of excision, which was comparable with the percentage contraction of betadine, a standard drug. Meanwhile, in the case of an *S. aureus*-infected wound, complete epithelization was observed in the 2% acetone-extract-treated group on the 12th day with a percentage wound contraction of 79.25% [46].

### 7.13. Photocatalytic Activity 

Methylene blue is a toxic cationic dye used to color paper, leather, and textiles. The dye in industrial effluent is mixed with river as well as other water sources, preventing solar radiation from penetrating and eventually affecting water-based photosynthesis, causing harm to the water ecology, environmental pollution, and poisoning the food chain [137]. Zinc-oxide nanoparticles obtained by green synthesis using fruit aqueous extract of *R. ellipticus* were found to degrade 17.5% of methylene blue within 1 h, with 72.7% of methylene dye degradation at the end of the experiment [138]. Likewise, Khalil et al. reported 98% of degradation of methylene blue dye after 150 minutes of exposure of *R. ellipticus*-leaf-mediated silver particles [139]. Thus, *R. ellipticus* is not only useful for its pharmacological properties but also showed a significant role (photocatalytic) in eliminating water pollution via dye degradation.

## 8. Conclusions and Future Prospective

*Rubus ellipticus* is a highly nutritious wild fruit with many health benefits including antioxidant, antidiabetic, anticancer, anti-inflammatory, nephroprotective, antipyretic, anticonvulsant, and anti-infective activities. Its fruits, leaves, and roots are rich in secondary metabolites like polyphenols, flavonoids, anthocyanins, tannins, and terpenoids. These findings in the literature validated the widespread use of this fruit in the folk medicinal system and the consumption of this wild fruit by local communities. Based on this evidence, this wild fruit and extract from different part of this medicinal plant hold strong potential for developing as a nutraceutical. Indeed, as this fruit is readily available in rural villages, it can also help to improve the nutritional status of people living in those areas and also uplift their economy, if properly utilized and marketed. Moreover, detailed research including focused bioactivity studies, standardizations of fruits from different climatic conditions, and advanced toxicological, pharmacological, and pharmacokinetic evaluations is necessary to determine additional uses and the impact of this fruit on the molecular level.

As the drug discovery research using herbal medicine and the demand of nutraceuticals continue growing, the future of wild nutritious fruits like *R. ellipticus* seems promising. This can play an important role as a source of lead compounds, herbal therapeutic agents, and functional foods. Moreover, this wildly growing medicinal plant can become a good source of nutrition and a means of economic upliftment for the people living in villages. So, future research should focus on confirming the scientific relevance of ethnomedicinal values, the mechanism of action on animal and human subjects, and the commercialization of this wild fruit. Such studies will help highlight this wild fruit and also establish good market value. Furthermore, as this fruit is merely collected from the wild, research on the cultivation and optimization of cultivation to obtain higher yield is also necessary to conserve this plant. This will ensure the sustainable use of this wild fruit and protect against overexploitation.

## Figures and Tables

**Table 2:** Macronutrient composition of fruit of *R. ellipticus* and *R. fruticosus*.

Nutrient Composition	Values for *R. ellipticus*	Values for *R. fruticosus*
Protein (%)	4.37 ± 0.52% [23]	1.39 g/100 g [90]
Fiber (%)	2.35 ± 0.05% [39]	44.2 % of dry matter [91]
Fat (%)	0.96 ± 0.20% [39]	0.49 g/100 g [90]
Carbohydrates (%)	86.4 ± 0.38% [23]	9.07 ± 0.80% [91]
Ash Value	2.97 g/100 g of dry matter [23]	3.0 g/100 g of fresh weight [92]

**Table 3:** Micronutrient composition of fruit of *R. ellipticus* and *R. fruticosus*.

Nutrient Composition	Quantity on *R. ellipticus*	Quantity on *R. fruticosus*
Sodium	89.43 ± 0.01 mg/100 g DW [23]	5.91 mg/100 g [93]
Potassium	1.82 ± 0.25 mg/100 g DW [39]	8.9 mg/g [31]
Calcium	0.95 ± 0.10 mg/100 g DW [39]	193mg/100 g [93]
Magnesium	118.72 ± 0.48 mg/100 g DW [23]	151 mg/100 g [93]
Copper	0.020 ± 0.01 mg/100 g DW [23]	165 µg/100 g [90]
Zinc	12.77 ± 0.05 mg/100 g DW [23]	2.3 mg/100 g [93]
Iron	4.249 ± 0.15 mg/100 g DW [23]	5.9 mg/100 g [93]
Manganese	1.948 ± 0.03 mg/100 g DW [23]	0.646 mg/100 g [90]
Ascorbic acid (Vitamin C)	19.8 mg/100 g Fresh weight [94]	7.1–9.6 mg/100 g [95]

DW: dry weight

## Data Availability

Not applicable.
